# *FURIN* variant associations with postexercise hypotension are intensity and race dependent

**DOI:** 10.14814/phy2.13952

**Published:** 2019-02-01

**Authors:** Burak T. Cilhoroz, Elizabeth D. Schifano, Gregory A. Panza, Garrett I. Ash, Lauren Corso, Ming‐Hui Chen, Ved Deshpande, Amanda Zaleski, Paulo Farinatti, Lucas P. Santos, Beth A. Taylor, Rachel J. O'Neill, Paul D. Thompson, Linda S. Pescatello

**Affiliations:** ^1^ Department of Kinesiology University of Connecticut Storrs Connecticut; ^2^ Department of Statistics University of Connecticut Storrs Connecticut; ^3^ Department of Preventive Cardiology Hartford Hospital Hartford Connecticut; ^4^ School of Nursing Yale University New Haven Connecticut; ^5^ Department of Physical Activity Sciences Rio de Janeiro State University Rio de Janeiro Brazil; ^6^ Department of Medical Sciences Federal University of Rio Grande do Sul Porto Alegre Brazil; ^7^ Institute for Systems Genomics University of Connecticut Storrs Connecticut; ^8^ Department of Molecular and Cell Biology University of Connecticut Storrs Connecticut

**Keywords:** Blood Pressure, exercise, hypertension, polymorphism

## Abstract

FURIN is a proprotein convertase subtilisin/kexin enzyme important in pro‐renin receptor processing, and *FURIN* (furin, paired basic amino acid cleaving enzyme) variants are involved in multiple aspects of blood pressure (BP) regulation. Therefore, we examined associations among *FURIN* variants and the immediate blood pressure (BP) response to bouts of aerobic exercise, termed *postexercise hypotension* (PEH). Obese (30.9 ± 3.6 kg^ ^m^−2^) Black‐ (*n* = 14) and White‐ (*n* = 9) adults 42.0 ± 9.8 year with hypertension (139.8 ± 10.4/84.6 ± 6.2 mmHg) performed three random experiments: bouts of vigorous (VIGOROUS) and moderate (MODERATE) intensity cycling and control. Subjects were then attached to an ambulatory BP monitor for 19 h. We performed deep‐targeted exon sequencing with the Illumina TruSeq Custom Amplicon kit. *FURIN* genotypes were coded as the number of minor alleles (#MA) and selected for additional statistical analysis based upon Bonferonni or Benjamini–Yekutieli multiple testing corrected *P*‐values under time‐adjusted linear models for 19 hourly BP measurements. After VIGOROUS over 19 h, as *FURIN* #MA increased in rs12917264 (*P* = 2.4E‐04) and rs75493298 (*P* = 6.4E‐04), systolic BP (SBP) decreased 30.4–33.7 mmHg; and in rs12917264 (*P* = 1.6E‐03) and rs75493298 (*P* = 9.7E‐05), diastolic BP (DBP) decreased 17.6–20.3 mmHg among Blacks only. In addition, after MODERATE over 19 h in *FURIN* rs74037507 (*P* = 8.0E‐04), as #MA increased, SBP increased 20.8 mmHg among Blacks only. Whereas, after MODERATE over the awake hours in *FURIN* rs1573644 (*P* = 6.2E‐04), as #MA increased, DBP decreased 12.5 mmHg among Whites only. *FURIN* appears to exhibit intensity and race‐dependent associations with PEH that merit further exploration among a larger, ethnically diverse sample of adults with hypertension.

## Introduction

Hypertension is the most prevalent cardiovascular disease (CVD) risk factor (World Health Organization, 2013, Benjamin et al. 2017). With the recently published American College of Cardiology (ACC)/American Heart Association (AHA) Guidelines for hypertension (Whelton et al. 2018). One hundred and three million (46%) adults in the United States now have hypertension compared to 72 million (32%) by the previous Joint National Committee 7 (JNC7) definition (Chobanian et al. 2003). The ACC/AHA guidelines emphasize the importance of lifestyle strategies such as exercise. Exercise lowers blood pressure (BP) 5–8 mmHg among adults with hypertension (Chobanian et al. 2003). These BP reductions are comparable in magnitude to first‐line antihypertensive medications and lower the risk of CVD 20–30% (Chobanian et al. 2003; Allhat 2002; Whelton et al. 2002).

Results from our group (Pescatello and Kulikowich 2001; Thompson et al. 2001) and others (Fitzgerald 1981; Wilcox et al. 1982; Haskell 1994; Halliwill 2001; Collier et al. 2008) indicate the chronic BP reductions that result from aerobic exercise training are related to the BP reductions following an acute aerobic exercise session, termed *postexercise hypotension* (PEH), that persists for 24 h after the exercise bout (Liu et al. 2012; Hecksteden et al. 2013; Tibana et al. 2015; Wegmann et al. 2017). Thus, PEH is a relevant clinical model to investigate the antihypertensive effects of aerobic exercise. However, 20–25% of adult do not exhibit PEH for reasons that are not clear that may be due, in part, to genetic predispositions (Ash et al. 2013a,b; Bruneau et al. 2016; Bouchard et al. 1995).

*FURIN* (furin, paired basic amino acid cleaving enzyme) consists of 16 exons and 15 introns on chromosome 15 band 15q26.1. Recent genome‐wide association studies with subjects of European descent demonstrated *FURIN* variants that included rs2521501, rs2071410, rs6227, and rs4702 are associated with elevated systolic BP (SBP) and diastolic BP (DBP) (Ehret et al. 2011; Ganesh et al. 2013; Turpeinen et al. 2015). The renin‐angiotensin‐aldosterone system (RAAS) is of key importance in the physiological and pathophysiological regulation of BP (Foëx and Sear 2004). Growing evidence shows FURIN, a proprotein convertase subtilisin/kexin enzyme, is directly involved in the modulation of the RAAS (Cousin et al. 2009) and sodium‐electrolyte balance (Schafer 2002; Hughey et al. 2004). FURIN participates in the enzymatic‐hormonal cascade of the RAAS by activating its key component, the pro‐renin receptor (PRR), which mediates the formation of angiotensin, a potent vasoconstrictor (Foëx and Sear 2004; Cousin et al. 2009; Schafer 2002; Hughey et al. 2004; Ren et al. 2017). In addition, FURIN is involved in the activation of an important renal sodium transporter, epithelial Na+ channel (ENaC), which is responsible for the rate limiting step of sodium reabsorption, a key factor for extracellular fluid volume and BP control (Schafer 2002; Hughey et al. 2004; Ren et al. 2017).

In previous work, we found that several variants in the RAAS associated with PEH in an intensity and race‐dependent manner. These renal variants included endothelial nitric oxide synthase (*NOS3*), angiotensin converting enzyme (*ACE),* angiotensin II (*AGT*), and angiotensin II type 1 receptor (*AGTR1*) (Augeri et al. 2009; Olson et al. 2012; Ash et al. 2013a; Pescatello et al. 2017). These findings and FURIN's regulatory role in BP control make *FURIN* a biological plausible candidate gene to explore for associations with PEH. Thus, the purpose of this study is to examine if *FURIN* variants exhibit intensity and race‐dependent associations with PEH among Black and White adults with hypertension.

## Material and Methods

### Overview

**Figure 1:**
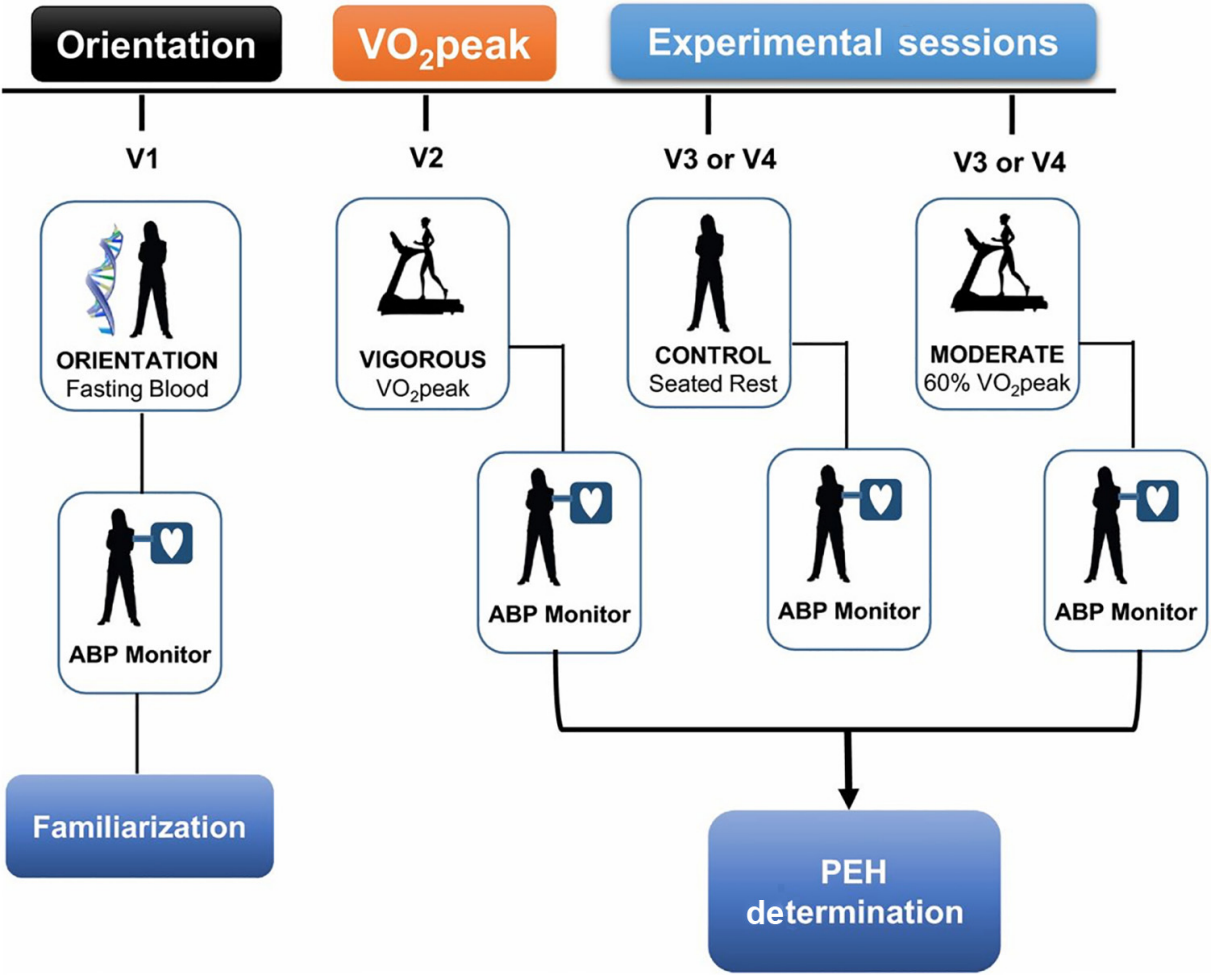
Study design overview. ABP, ambulatory blood pressure worn until the next morning; *V*O_2peak_, peak oxygen consumption as determined on the peak cardiopulmonary graded exercise stress test.

We applied the same study design used in our previous PEH studies as illustrated in Figure 1 (Blanchard et al. 2006; Pescatello et al. 2008; Augeri et al. 2009; Olson et al. 2012; Ash et al. 2013a; Pescatello et al. 2016, 2017). The subjects and data from two previous reports (Pescatello et al. 2016, 2017) were used in the current study as well. We obtained a blood sample for deep‐targeted exon sequencing and a fasting cardiometabolic profile at the orientation session. The subjects then left the laboratory wearing an ambulatory BP monitor until the next morning to familiarize them with the technology (Pescatello et al. 2017; Ash et al. 2013a,b). Subsequently, the subjects underwent three randomly assigned acute experiments: a cardiopulmonary graded exercise test (GEST) on a cycle ergometer to measure peak oxygen consumption (*V*O2peak) (VIGOROUS); 30 min of cycling at 60% *V*O_2peak_ (MODERATE); and a 30‐min control session of seated rest (CONTROL). We measured BP for 20 min before and 45 min after these experiments. Subjects then left the laboratory wearing an ambulatory BP monitor for 19 h until the next morning.

### Subjects

Subjects (*n* = 23) were 18–55 years of age. They were sedentary (i.e., exercising ≤2 days per week), and overweight to obese [i.e., body mass index (BMI) ≥25 to <40 kg m^−2^] with elevated to stage 1 hypertension (Whelton et al. 2018) (Table 1). They self‐reported their race as White or Black. Participants were required to stop using any medications (e.g., oral steroids, aspirin, herbal supplements, etc.) that could possibly influence BP at least 4 weeks prior to testing. Subjects with any musculoskeletal impairments were not enrolled if these conditions restricted their ability to complete the exercise experiments. With physician permission, two subjects stopped using their antihypertensive medications ≥ 6 weeks prior to study participation. Women had regular menstrual cycles. The subjects’ weight was monitored throughout study duration to ensure weight stability (i.e., gaining or losing <2.25 kg of baseline body weight). All subjects completed an informed consent approved by the Institutional Review Boards of the University of Connecticut and Hartford Hospital.

**Table 1:** Subject Characteristics (X ± SD) (Pescatello et al. 2016)

Variable	Whites (*n* = 9)	Blacks (*n* = 14)
Age (year)	45.1 ± 7.8	39.9 ± 10.6
Gender (male/female)	9/0	10/4
Body mass index (kg m^−2^)	30.5 ± 1.8	31.1 ± 4.5
Waist circumference (cm)	98.0 ± 7.2	88.3 ± 9.4^1^
Relative peak oxygen consumption (mL kg^−1^ min^−1^)	29.7 ± 6.4	25.3 ± 5.7
Awake systolic blood pressure (mmHg)	139.3 ± 7.0	140.2 ± 12.3
Awake diastolic blood pressure (mmHg)	85.0 ± 5.1	84.3 ± 6.9
Glucose (mg dL^−1^)	96.4 ± 12.2	97.2 ± 10.3
Ambulatory arterial stiffness index	0.415 + 0.93	0.391 + 0.143
Insulin (*μ*lU mL^−1^)	13.1 ± 10.1	9.4 ± 6.0
Homeostatic Model of Assessment	3.1 ± 2.2	2.3 ± 1.5
Total cholesterol (mg dL^−1^)	207.8 ± 31.3	178.5 ± 27.3^1^
Low density lipoproteins (mg dL^−1^)	129.4 ± 20.3	108.3 ± 32.7
High density lipoproteins (mg dL^−1^)	44.1 ± 10.9	53.9 ± 14.8
Triglycerides (mg dL^−1^)	170.8 ± 88.8	83.7 ± 35.8^2^
Nitrite (NO_2_^−^)/Nitrate (NO_3_^−^) (*μ*mol L^−1^)	23.3 ± 37.0	10.9 ± 13.1
C‐reactive protein (mg dL^−1^)	1.1 ± 1.0	2.8 ± 3.5
Endothelin (pmol L^−1^)	0.222 ± 0.213	0.378 ± 0.663
Plasma renin activity (ng mL^−1^ h^−1^)	1.7 ± 1.09 (*n* = 2)	0.946 ± 0.840 (*n* = 8)

1 *P *<* *0.05.

2 *P *<* *0.01.

### Body composition

Body mass index (BMI) (kg m^−2^) was calculated from body weight and height with a calibrated balance beam scale. Waist circumference was measured with a non‐flexible Gulick tape measure at the narrowest part of the torso (Pescatello et al. 2013).

### Blood pressure

At the orientation session, BP was measured with standard procedures (Pickering et al. 2006) using an automated BPTRU monitor (BPTRU Medical Devices; Coquitlam, Canada) to identify BP status. Before the experiments, all participants remained seated for 20 min, and BP was measured every 2 min in the non‐dominant arm with the automated BPTRU monitor. The BP measurements taken in the last 10 min of the baseline period were averaged and recorded as baseline BP. After the orientation session and the three experiments (i.e., CONTROL, MODERATE, VIGOROUS) following our previous protocol (Augeri et al. 2009; Olson et al. 2012; Ash et al. 2013a; Pescatello et al. 2017) subjects were attached to an ambulatory BP monitor (Oscar2 automatic noninvasive ambulatory BP monitor, Suntech Medical Instruments Inc., Raleigh, NC) with a calibration check done by a mercury sphygmomanometer. After the 45‐min postexercise recovery period, the ambulatory BP monitor was programmed to obtain three ambulatory BP assessments for each waking hour and two for each sleeping hour over 19 h. We instructed the subjects to proceed with their normal daily activities and not to engage in formal exercise while wearing the monitor. They noted any unusual physical or emotional events as well as their awake (*n* = 10 h) and sleep (*n* = 9 h) time to total 19 h of wearing the monitor on a journal diary that they carried with themselves while wearing the ABP monitor. We removed ambulatory BP readings of systolic BP (SBP) >220 or <80 mmHg or diastolic BP (DBP) >130 or <40 mmHg according to the manufacturer's exclusion criteria. Ambulatory BP reports were valid if we received at least 80% of the potential BP readings. We calculated the ambulatory arterial stiffness index (AASI) after CONTROL as 1–(slope of DBP vs. SBP over 19 h.) (Dolan et al. 2006).

### Acute experiments

Subjects finished three randomly assigned acute experiments: a non‐exercise control session of seated rest (CONTROL) and two cycle exercise bouts on an upright cycle ergometer (Monarch 839E Digital Cycle Ergometer, Stockholm, Sweden) at 60% (MODERATE) and 100% (VIGOROUS) *V*O_2peak_ (Fig. 1). The three experiments were separated by a minimum of 48 h to avoid the confounding effects of acute exercise and performed at the same time of day to account for the diurnal variation in BP. The participants were instructed to consume a standard breakfast 2–3 h before and refrain from caffeinated beverages for 6 h before all experiments. All experiments for a given subject were completed within 1 month of beginning the study.

The same research assistant measured heart rate (HR), SBP, and DBP for all subjects and experiments. At the beginning of each experiment subjects were seated at rest for a 20‐min baseline period during which HR was recorded with a HR monitor (Polar Electro Inc., Port Washington, NY) every 2 min, while SBP and DBP were obtained every other minute by auscultation. BP and HR were measured in the seated position every 2 min during the 45‐min postexercise recovery period. The subjects left the laboratory wearing an ambulatory BP after the experiments until they woke the following morning when they detached the monitor and physically returned it along with the journal to the research assistant. The research assistant examined the journal and ABP reports after each visit for any unusual physical or emotional events that may have impacted a reading.

VIGOROUS (100% *V*O_2peak_) consisted of a peak graded cardiopulmonary GEST on a cycle ergometer of continuous cycling at a constant cadence (60 rev/min) starting with a resistance of 0.5 kp (30 W) that was increased 0.5 kp every 2 min until volitional exhaustion. *V*O_2peak_ was measured by breath‐by‐breath analysis of expired gases (ParvoMedicsTrueOne^®^ 2400 Metabolic Measurement System, ParvoMedics Inc., Sandy, UT). HR was recorded continuously with a 12‐lead electrocardiograph (Marquette Case 8000, Jupiter, FL), and BP was obtained every 2 min by auscultation during the GEST. The workload of the other exercise experiment (MODERATE) was calculated using the results of the peak cardiopulmonary GEST (VIGOROUS). The subjects performed the two remaining experiments in random order: non‐exercise control of 30 min of seated rest and MODERATE (60% *V*O_2peak_) which consisted of a 5‐min warm up of cycling with no resistance, 20 min of cycling at 60% *V*O_2peak_, and a 5‐min cool down to total 30 min. HR, SBP, and DBP were measured at 5‐min intervals during non‐exercise control and MODERATE.

### Blood sampling and analysis

Fasting blood samples were drawn without stasis from an antecubital vein and centrifuged at 3400*g* at 23°C for 10–15 min during the orientation session. Serum was separated into red top and plasma samples in EDTA vacutainer tubes. Serum and plasma samples were pipetted into different 1.8 mL non‐pyrogenic storage tubes and frozen at −80°C for future analysis. Enzymatic/spectrophotometric methods were used to identify glucose and insulin and calculate the homeostasis model assessment, a biomarker of insulin resistance (Matthews et al. 1985). The same methods were used to determine total cholesterol, triglyceride, and high‐density lipoprotein cholesterol concentrations from which low‐density lipoprotein cholesterol was calculated with the Friedewald equation (Friedewald et al. 1972). Nitrite (NO_2‐_)/Nitrate (NO_3‐_), high sensitivity C‐reactive protein (CRP), endothelin 1–21, and plasma renin activity (PRA) were also measured by enzymatic/spectrophotometric methods. Blood analyses were done with two levels of quality control. A blood sample for DNA analysis was drawn into an EDTA purple top vacutainer tube that was centrifuged for white cell isolation and frozen at −80°C for future DNA extraction.

### Targeted sequencing and variant calling

Using the Illumina TruSeq Custom Amplicon kit (Catalog# FC‐130‐1001, Illumina, San Diego, CA) (Pescatello et al. 2017), we carried out deep‐targeted exon sequencing of a prioritized panel of 41 genes. This panel contained polymorphisms reported to be associated with hypertension, the BP response to pharmacotherapy, and/or the BP response to PEH and exercise training (Ash et al. 2013a; Bruneau et al. 2016). The Illumina Design Studio software was used to create probes for the generation of 1214 amplicons with a size range of 225–275 bp. The TruSeq Custom Amplicon file included Target ID, region, chromosome, and start and end hg19 reference coordinate positions. Following the TruSeq Custom Amplicon Library Preparation Guide, sequencing libraries were prepared. For all libraries, DNA input mass was 250 ng of DNA. We generated libraries with dual indices (23 PCR cycles) and then completed normalization and pooling. Illumina MiSeq version 2 reagents (250 paired‐end reads) were used to sequence the library amplicon pool. From the library amplicon pool, 7.1 million pair‐end reads (6.8 million passing quality filter) were obtained. MiSeq Reporter Software (version 2.3.32), using the TruSeq Amplicon workflow, created Fastq files and aligned reads to the hg19 human reference sequence with the Smith‐Waterman algorithm. The Genome Analysis Toolkit (GATK) was used for variant calling (single‐nucleotide variants and small insertion/deletions) and the formation of the variant calling files (VCF). For all further downstream analysis, a merged VCF was formed with VCF tools v 0.1.12b (Danecek et al. 2011) and custom R scripts (R v3.2.0). Only variants with FILTER = PASS were kept. We calculated the total number of variants present per subject and each polymorphism's major and minor allele (MA) frequency for each defined amplicon target region of each subject.

### Statistical analysis

Descriptive statistics (Mean ± SD) were performed for the total sample and by racial group. We utilized independent *t*‐tests to determine if there were differences in subject descriptive characteristics by racial group. Moreover, we used repeated measures analysis of covariance to test if the BP response, defined as the change from baseline following MODERATE, VIGOROUS, and CONTROL at hourly intervals under ambulatory conditions, differed over 19 h with age and BMI as covariates and gender and race as fixed factors. We used SPSS 14.0 (Chicago, IL) for statistical analysis.

### Variant screening

The screening technique has been described in detail previously (Pescatello et al. 2016, 2017). Briefly, we analyzed genotypic values coded as the number of MA (#MA) for 645 variants from 41 genes (Pescatello et al. 2016, 2017). Linear r mixed models were fit for each race separately that included polynomial time (order 3), polymorphism under an additive model, and polymorphism x time interactions as covariates, BP response as the dependent variable, and a first‐order autoregressive (AR1) correlation structure for the 19 repeated observations within subject. For each polymorphism, Bonferroni and Benjamini–Yekutieli (BY) (Benjamini and Yekutieli 2005) adjusted *P*‐values were calculated, correcting for the total number of unique polymorphism profiles among each racial group resulting in 300 polymorphisms for Blacks and 146 for Whites. Similar models were fit over the 10 h awake period. *FURIN* polymorphisms that passed the multiple testing thresholds (i.e., Bonferroni adjusted *P* < 0.05 and/or BY adjusted *P*‐values) were examined for further statistical modeling and analysis. *FURIN* genotype‐BP differences by the #MA after VIGOROUS and MODERATE compared to CONTROL were presented as the average change over time with the associated *P* value resulting from the screening model that accounted for repeated measures over time.

### Final multivariable regression models

As stated in our previous reports (Pescatello et al. 2016, 2017), for each polymorphism passing the multiple testing threshold, covariates were chosen for inclusion in the final models if they were marginally associated (*P* < 0.05) with the BP response using models fit by maximum likelihood (ML). AR1 provided the best fit in terms of Akaike Information Criteria in all cases compared to other possible within subject correlation structures (e.g., compound symmetry and independent structures). We report the likelihood ratio test (LRT) *P*‐values and the partial proportion of variation explained (PVE) for the polymorphism effects based on ML estimation using AR1 within‐subject correlation structure. The PVE for a given model was defined as *R* ^2 ^_m _= 1‐(*L*_R_/*L*_U_)^2/*n*^, where *L*_R_ is the restricted maximized likelihood from a model containing only an intercept, *L*_U_ is the unrestricted maximized likelihood for the given model, and *n* is the number of subjects (Maddala 1983; Magee 1990). The partial PVE for each polymorphism was the difference between *R* ^2^_m_ for the final model (including the covariate and polymorphism effects) and *R* ^2^_m_ for the final model excluding polymorphism effect(s) (Schemper 1993). The parameter estimates for the final models using the restricted maximum likelihood (REML) method are also reported. Statistical analysis for screening and final models was performed in *R* and SAS version 9.4, respectively.

### Annotation assessment of causal variation and regulatory effects

For each polymorphism that passed the multiple testing threshold, we determined the combined annotation‐dependent depletion (CADD) score (Kircher et al. 2014) that quantitatively prioritizes functional, deleterious, and disease causal variants across a wide range of functional categories, effect sizes, and genetic architectures. More severe allelic substitutions in terms of their causal variation correspond to higher CADD scores. CADD scores of ≥10 indicate that substitutions in a polymorphism are predicted to be the 10% most deleterious substitutions in the human genome. Moreover, ENCODE‐based data sets were searched to derive regulatory effects for the polymorphisms that passed the multiple testing threshold using the Chromatin Immunoprecipitation Coupled to Massively Parallel Sequencing (ChIP‐seq) annotations across ENCODE cell lines and the human reference lymphoblast cell line GM12878 hg19. Genome segmentation data for hg19 was based on 25 different chromatin states (including eight different chromatin marks, RNA polymerase II, and CTCF binding) that are used to segment the genome using both ChromHMM and Segway (Hoffman et al. 2012).

## Results

### Subjects

Subject characteristics for all variables have been previously reported (Pescatello et al. 2016, 2017) and are shown in Table 1. There were 19 men, 10 of who were black and nine white, and four black women that participated who on average were middle‐aged and of poor to very poor cardiorespiratory fitness for men and women of their age (Pescatello et al. 2013). Although the current study examined variants in a different gene from our previous reports (Pescatello et al. 2016, 2017), the subjects and BP phenotype examined were the same across these reports. Accordingly, when the minor allele counts were similar for the *FURIN* variants in a given subject as in the different genetic variants in our previous reports, the covariate values and linear mixed models regarding the BP results are similar across the three studies.

### The blood pressure response by number of FURIN variant minor alleles

**Figure 2:**
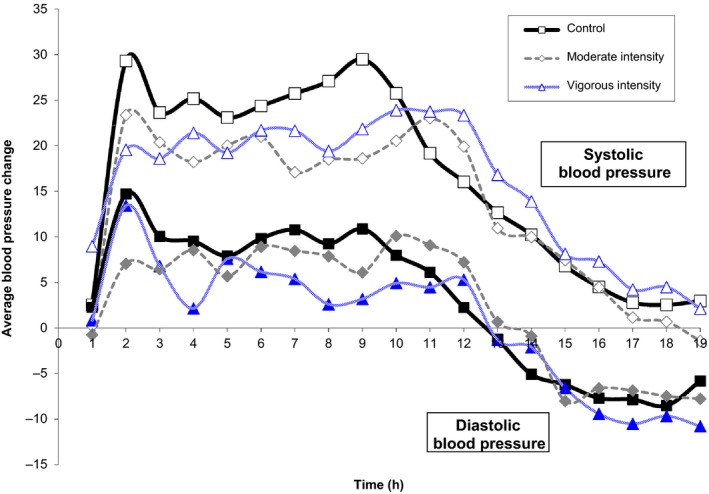
Overall blood pressure (BP) change. The average change in ambulatory systolic and diastolic BP from baseline after aerobic exercise versus control at hourly intervals over 19 h. CONTROL, nonexercise session of seated rest; MODERATE, 60% *V*O_2peak_; *P* > 005 exercise versus nonexercise control (Pescatello et al. 2016).

The SBP and DBP responses did not differ among the total sample (Fig. 2) (Pescatello et al. 2016, 2017). Similar to our previous findings for *ACE, AGTR1, CYP11B2, ADD1* (Pescatello et al. 2016), and *NOS3* (Pescatello et al. 2017), differences were found between Whites and Blacks for SBP and DBP responses over 19 h after VIGOROUS and MODERATE compared to CONTROL by *FURIN* variant #MA passing multiple testing thresholds.

For *FURIN* rs12917264 (*P* = 2.4E‐04) and rs75493298 (*P* = 6.4E‐04), systolic BP (SBP) decreased 30.4–33.7 mmHg. For *FURIN* rs12917264 (*P* = 1.6E‐03) and rs75493298 (*P* = 9.7E‐05), diastolic BP (DBP) decreased 17.6–20.3 mmHg. In addition, for *FURIN* rs74037507 (*P* = 8.0E‐04), SBP increased 20.8 mmHg among Blacks only. Whereas, after MODERATE over the awake hour, as the #MA increased for *FURIN* rs1573644 (*P* = 6.2E‐04), DBP decreased 12.5 mmHg among Whites only (Table 2).

**Table 2:** Blood pressure response (X ± SD)^1^ after versus before VIGOROUS and MODERATE compared to control over 19 hours (rs12917264, rs75493298, and rs74037507) and AWAKE (rs1573644) by *FURIN* # of Minor Alleles Located on Chromosome (Chr) chr15:90,868,592‐90,883,458.

				Racial/Ethnic Group					
				Blacks (*n* = 14)			Whites (*n* = 9).		
Variant		Regulatory Effects^2^	CADD Score	# Minor Alleles	SBP (mmHg)	DBP (mmHg)	# Minor Alleles	SBP	DBP
VIGOROUS	rs12917264	Transcript Variant in an Intron Enhancer/Promotor (K562 cells) Predicted transcribed in GM 12878	3.211	0 (*n* = 13) 1 (*n* = 1) 2 (*n* = 0)	**0.8 **±#x00A0;13.1 **−32.8 ±** ** NA** **NA**	**0.4 **±** 8.1** **−17.2 **±** NA** **NA**	0 (*n* = 9) 1 (*n* = 0) 2 (*n* = 0)	0.5 ± 10.1 NA NA	−0.1 ± 5.3 NA NA
	rs75493298	Transcript Variant in an Intron Overlaps 19 transcripts and 1 regulatory feature	1.978	0 (*n* = 13) 1 (*n* = 0) 2 (*n* = 1)	**0.6 **±** 13.7** **NA** −**29.8 **±** NA**	**0.6 **±** 7.6** NA **−19.7 **±** NA**	0 (*n* = 8) 1 (*n* = 1) 2 (*n* = 0)	0.4 ± 10.8 1.6 ± NA NA	0.0 ± 5.8 −1.9 ± NA NA
MODERATE	rs74037507	5′ Upstream Gene Variant Predicted Transcribed in K562 cells	3.634	0 (*n* = 13) 1 (*n* = 1) 2 (*n* = 0)	−**4.7 **±** 7.3** **16.1 **±** NA** **NA**	−3.5 ± 5.9 3.9 ± NA NA	0 (*n* = 9) 1 (*n* = 0) 2 (*n* = 0)	−3.0 ± 6.6 NA NA	−1.6 ± 5.2 NA NA
	rs1573644^3^	Alternate Transcript Variant in an Intron Same Enhancer/Promoter Block as rs2071410 (K562 cells) Predicted Repressed/Low Activity in GM 12878	6.988	0 (*n* = 10) 1 (*n* = 3) 2 (*n* = 1)	−3.8 ± 11.5 −4.6 ± 6.5 −22.9 ± NA	−3.8 ± 8.5 −2.2 ± 4.7 −10.8 ± NA	0 (*n* = 3) 1 (*n* = 3) 2 (*n* = 3)	−0.9 ± 1.3 −9.1 ± 10.5 −9.8 ± 0.7	**1.4 ± 1.1** −**2.5 ± 5.8** −**11.1 **±** 5.5**

SBP, Systolic Blood Pressure; DBP, Diastolic Blood Pressure; K562, myelogenous leukemia line cells; GM12878, Lymphoblastoid cell line; H1ES; Hexaploid H1 (ES).

1 *X* and SD are computed as the average and standard deviation, respectively, of the subject‐level BP response averaged over 19 h. Bolded values correspond to significant effects after multiple testing adjustment.

2 Chromosome location and function obtained from SNAP, SNP Annotation and Proxy Search www.broadinstitute.org/mpg/snap/ldsearch.php; University of California Santa Cruz UCSC Genome Brower http://www.genome.ucsc.edu/; and Ensembl: http://grch37.ensembl.org/index.html; CADD, Combined Annotation Dependent Depletion, a score that prioritizes causal variation and regulatory effects http://cadd.gs.washington.edu/score.

3 rs1573644, rs2071410, and rs6227 were significantly associated with the same magnitude of DBP decrease after MODERATE over the awake among only Whites, but we chose to report rs1573644 due to its higher CADD score.

### Proportion of variance explained

Table 3 shows the PVE in the multivariable regression models for the SBP and DBP response to VIGOROUS and MODERATE among Blacks, with and without FURIN polymorphisms. Resting ambulatory SBP over 19 h, age, resting AASI over 19 h, and time (order 3) accounted for 92.5% of the variation in the SBP response to VIGOROUS. Furthermore, the individual FURIN variants explained up to 5.6% (*P* = 6.3E‐04) of the variation when resting ambulatory SBP over 19 h, resting AASI over 19 h, and age in the model were accounted for. For the DBP response to VIGOROUS, fasting triglycerides, gender, and endothelin accounted for 85.8% of the variation. However, the individual FURIN variants explained up to 7.6% (*P* = 1.1E‐03) of the variation in the DBP response to VIGOROUS, when fasting triglycerides, gender, and endothelin in the model were accounted for. For the SBP response to MODERATE, insulin accounted for 66.2% of variation. When insulin was accounted for, FURIN variants explained an additional 6.9% (*P* = 0.0736) of the variations in the SBP response to MODERATE.

**Table 3:** Proportion of variance explained in the multivariable regression models for the systolic and diastolic blood pressure response following VIGOROUS and MODERATE among Blacks, with and without polymorphisms from the *FURIN* gene

VIGOROUS			
** **SBP			
Polymorphism	Model^1^	PVE	*P* value^3^
None	BP Response = −1.7872 + 2.1590*time + 0.0207*time^2−0.0320*time^3 + 16.4343*log(AASICONT) + 0.6782*Orientation 19 h SBP + 0.5765*Age	0.9252	–
Polymorphism	Model^1^	Partial PVE^2^	*P* value^3^
rs12917264	BP Response = −0.1207 + 2.1486*time + 0.0149*time^2 −0.0318*time^3 + 15.8916*log(AASICONT) + 0.6262*Orientation 19 h SBP + 0.3839*Age −21.3422*SNP	0.0358	0.0026
rs75493298	BP response = −0.2841 + 1.5349*time + 0.0091*time^2 − 0.0210*time^3 + 11.1560*log(AASICONT)+ 0.6298*Orientation 19 h SBP + 0.5368*Age – 9.2927*SNP + 4.2776*SNP*time + 0.0309*SNP*time^2 − 0.0753*SNP*time^3	0.0562	6.27 × 10^−4^
DBP			
Polymorphism	Model*	PVE	*P* value^3^
None	BP Response = −3.1187−4.3889*log(Endothelin)−8.5055*log (TRIG) + 7.6938*Gender	0.8577	–
Polymorphism	Model*	Partial PVE^2^	*P* value^3^
rs12917264	BP Response = −2.9066−4.1740*log(Endothelin)−8.5088*log(TRIG) + 7.3595*Gender−1.6472*SNP	0.0010	0.7518
rs75493298	BP Response = −1.7776−4.0777*log(Endothelin)−5.5915*log(TRIG) + 6.8446*Gender−7.6246*SNP	0.0760	0.0011
MODERATE			
SBP			
Polymorphism	Model*	PVE	*P* value^3^
None	BP Response = −3.2665 + 10.9273*log(INSULIN)	0.6623	–
Polymorphism	Model*	Partial PVE^2^	*P* value^3^
rs74037507	BP Response = −4.1181 + 8.0972*log(INSULIN) + 11.9101*SNP	0.0690	0.0736

VIGOROUS, 100% of peak oxygen consumption (*V*O_2peak_); MODERATE, 60% *V*O_2peak_; SBP, systolic blood pressure; DBP, diastolic blood pressure; PVE, proportion of variance explained; SNP, polymorphism; TRIG, triglycerides; AASICONT, resting ambulatory arterial stiffness index over 19 h; Orientation 19 h; SBP, resting ambulatory SBP over 19 h.

1 Restricted maximum likelihood estimates reported; all covariates centered except for polymorphism.

2 Either polymorphism only (when there is no polymorphism by time interaction) or joint polymorphism and polymorphism by time effects (when there is a polymorphism by time interaction), computed using maximum likelihood.

3 Likelihood ratio tests for either polymorphism only (when there is no polymorphism by time interaction) or joint polymorphism and polymorphism by time effects (when there is a polymorphism by time interaction) under maximum likelihood.

Table 4 shows the PVE for the DBP response to MODERATE among Whites for the final multivariable regression models without the *FURIN* variants and the partial PVE for each *FURIN* polymorphism after accounting for the other covariates in the model. Covariates including CRP and family history of hypertension accounted for 75.8% of the variation in DBP response to MODERATE. Whereas, the individual *FURIN* variants explained up to 15.0% (*P* = 7.75E‐04) of the variation in the DBP response to MODERATE, when the other covariates in the model were accounted for.

**Table 4:** Proportion of variance explained in the multivariable regression models for the diastolic blood pressure response following MODERATE among Whites, with and without polymorphisms from the *FURIN* gene

MODERATE			
DBP			
Polymorphism	Model^1^	PVE	*P* value^3^
None	BP Response = 4.1561 + 5.0085*log(CRP) ‐7.4992FxHTN	0.7588	–
Polymorphism	Model^1^	Partial PVE^2^	*P* value^3^
rs1573644	BP Response = 5.5691 + 3.4873*log(CRP)−5.8225FxHTN−3.7874*SNP	0.1519	7.75 × 10^−4^
rs1573644^4^	BP Response = 3.4027 + 2.7870*log(CRP) ‐7.1663FxHTN−6.7335*SNPr	0.1439	0.0013

MODERATE, 60% *V*O_2peak_; DBP, diastolic blood pressure; PVE, proportion of variance explained; SNP, polymorphism; CRP, C Reactive Protein; FxHTN, Family History of Hypertension.

1 Restricted maximum likelihood estimates reported; all covariates centered except for polymorphism.

2 Either polymorphism only (when there is no polymorphism by time interaction) or joint polymorphism and polymorphism by time effects (when there is a polymorphism by time interaction), computed using maximum likelihood.

3 Likelihood ratio tests for either polymorphism only (when there is no polymorphism by time interaction) or joint polymorphism and polymorphism by time effects (when there is a polymorphism by time interaction) under maximum likelihood.

4 Recessive model for polymorphism (i.e., SNPr = 0 if 0 or 1 copies of the minor allele; SNPr = 1 if 2 copies of the minor allele; additive genetic models used for all other polymorphisms (i.e., SNP = #minor allele).

## Discussion

In this study, we investigated whether *FURIN* variants exhibited significant associations with PEH among Black and White adults with elevated BP to Stage 1 hypertension. Our findings revealed that *FURIN* rs12917264, rs75493298, rs1573644, and rs74037507 significantly associated with PEH in an intensity and racially dependent manner. For *FURIN* rs12917264 and rs75493298, after VIGOROUS over 19 h as the #MA increased, SBP *decreased* by 30–34 mmHg and DBP *decreased* 17–20 mmHg after VIGOROUS compared to CONTROL among Blacks only. Additionally, for *FURIN* rs1573644, after MODERATE over the awake hour, as the # MA increased, DBP *decreased* 12.5 mmHg among Whites only. In contrast, for *FURIN* rs74037507, as the #MA increased, SBP *increased* 20.8 mmHg after MODERATE over 19 h among Blacks only.

The BP reductions we observed in the *FURIN* genetic variants of rs12917264, rs75493298, and rs1573644 is comparable to that seen with antihypertensive medication use (Chobanian et al., 2003) and sufficient to lower elevated BP unto normal BP ranges for the remainder of the day following a bout of acute exercise. Furthermore, these findings are consistent with our previous studies investigating *NOS3* and renal variants that included *ACE*,* AGT*,* AGTR1, CYP11B2* (Augeri et al. 2009; Olson et al. 2012; Ash et al. 2013a; Pescatello et al. 2017). Overall, these *FURIN* variants accounted for 3.6–15.2% of the variance in BP response to VIGOROUS or MODERATE. The magnitude of this variation explained appears to be larger than that reported for individual variants in previous exercise studies investigating health‐related phenotypes (Olson et al. 2012; Bruneau et al. 2016). On the other hand, we found the clinical biomarkers that included arterial stiffness index, age, gender, fasting insulin, triglycerides, endothelin, family history, and CRP accounted for 66.2–92.5% of the variation in the BP response after VIGOROUS or MODERATE depending on the final multivariable regression model. These findings are consistent with a provocative recent study (Pazoki et al. 2018) that found traditional clinical biomarkers of CVD risk that included healthy lifestyle behaviors such as physical activity, have stronger effects on resting BP levels than do genetic predispositions.

While high BP affects different racial/ethnic populations, the prevalence of hypertension in Blacks is the highest among all ethnic groups at 59% among men and 56% among women according to the BP classification scheme of the new ACC/AHA Guidelines for hypertension (Whelton et al. 2018). Recently, Diaz et al. (2017) have shown regular participation in vigorous intensity physical activity has an important role in reducing incident hypertension, and thereby, improving cardiovascular health among Blacks. Similarly, Liu et al. (2017) found that blacks had a larger reduction in incident hypertension than whites of 24% versus 6%, respectively, when meeting or exceeding the physical activity recommendations. Our findings support these studies in that vigorous physical exercise confers the largest BP reductions among Blacks who were FURIN variant MA carriers, a finding consistent with our previous reports from the same dataset (Pescatello et al. 2016, 2017).

Several lines of evidence showed that racial differences exist in PEH between Blacks and Whites for reasons that are not clear (Headley et al. 1998; Pescatello et al. 2003; Santa‐Clara et al. 2003; Bond et al. 2005; Brandon and Elliott‐Lloyd 2006; Jones et al. 2006, 2007; Enweze et al. 2007; Yan et al. 2016). Nonetheless, the role of *FURIN* in the modulation of PEH is supported by its important BP regulatory actions. FURIN, a proprotein convertase subtilisin/kexin enzyme, participates in the regulation of extracellular fluid volume and angiotensin formation via the activation of the ENaC and PRR in the RAAS (Cousin et al. 2009; Schafer 2002; Hughey et al. 2004). Overactivation of ENaC results in increased salt and water retention (Lindhorst et al. 2007) and is thought to arise from *ENaC* mutations (Lindhorst et al. 2007; Baker et al. 1998) that have been shown to be far more prevalent in Blacks with hypertension compared to Blacks with normal BP (Baker et al. 1998; Henderson et al. 2004). Furthermore, overexpression of PRR leads to an enhanced aldosterone secretion stimulated by excess angiotensin production, promoting incident hypertension (Burckle et al. 2006; Yang 2015; Xu et al. 2016).

Moreover, recent genome‐wide association studies (Ehret et al. 2011; Ganesh et al. 2013; Turpeinen et al. 2015) revealed that *FURIN* rs4702, rs6227, rs2521501, and rs2071410 were significantly associated with elevated BP after adjustment for multiple testing. Of note, *FURIN* rs2071410 (*r* ^2 ^= 0.927), rs6227 (*r* ^2 ^= 0.927), and rs1573644 (*r* ^2 ^= 0.857) that were significantly associated with PEH in our study were in strong linkage disequilibrium with *FURIN* rs2521501 that was observed by Ehret et al. (2011), reinforcing the role of *FURIN* in BP regulation. In addition, Turpeinen et al. (2015) showed that *FURIN* rs4702 strongly associated with systemic vascular resistance (*P* = 0.005) but not cardiac output (*P* = 0.09). It is well‐established that PEH occurs due to reductions in systemic vascular resistance with no concomitant increase in cardiac output among adults with hypertension who are healthy other than their hypertension such as the subjects in our study (Halliwill 2001). Therefore, the intensity and racial dependent modulations of PEH by *FURIN* variants may be due to alterations in systematic vascular resistance via its regulatory actions on extracellular fluid volume and angiotensin formation via the RAAS.

The methods used in our power calculations are described in detail elsewhere (Pescatello et al. 2016, 2017). Using these methods, we calculated the power to detect the BP‐*FURIN* genotype significant differences ranged from 11% for rs75493298 to 40% for rs12917264 in Table 2. To the best of our knowledge, this study is the first reporting that *FURIN* variants exhibited exercise intensity and racial dependent associations with PEH. Nonetheless, the small sample size is a major limitation. Thus, our preliminary findings from these models should be interpreted cautiously. Future mechanistic studies are needed to confirm our novel findings among a larger, ethnically diverse sample of men and women with hypertension to better establish the role of *FURIN* variants in the regulation of the BP response to both acute (i.e., PEH) and chronic (i.e., training) aerobic exercise.

## Conflict of Interest

None declared.
